# Clinical and cost-effectiveness of bracing in symptomatic knee osteoarthritis management: protocol for a multicentre, primary care, randomised, parallel-group, superiority trial

**DOI:** 10.1136/bmjopen-2020-048196

**Published:** 2021-03-26

**Authors:** Melanie A Holden, Michael Callaghan, David Felson, Fraser Birrell, Elaine Nicholls, Sue Jowett, J Kigozi, John McBeth, Belinda Borrelli, Clare Jinks, Nadine E Foster, Krysia Dziedzic, Christian Mallen, Carol Ingram, Alan Sutton, Sarah Lawton, Nicola Halliday, Liz Hartshorne, Helen Williams, Rachel Browell, Hannah Hudson, Michelle Marshall, Gail Sowden, Dan Herron, Evans Asamane, George Peat

**Affiliations:** 1 Primary Care Centre Versus Arthritis, School of Medicine, Keele University, Keele, UK; 2 Faculty of Health, Psychology & Social Care, Manchester Metropolitan University, Manchester, Greater Manchester, UK; 3 Boston University School of Medicine, Boston, Massachusetts, USA; 4 Research in OsteoArthritis Manchester (ROAM), Centre for Epidemiology Versus Arthritis, Centre for Musculoskeletal Research, Institute of Inflammation and Repair, The University of Manchester, Manchester, Manchester, UK; 5 Medical Research Council Versus Arthritis Centre for Integrated Research into Musculoskeletal Ageing, Newcastle University, Newcastle upon Tyne, Tyne and Wear, UK; 6 Northumbria Healthcare NHS Foundation Trust, North Shields, Tyne and Wear, UK; 7 Clinical Trials Unit, Keele University, Keele, Staffordshire, UK; 8 Health Economics Unit, University of Birmingham, Birmingham, UK; 9 Faculty of Biology, Medicine and Health, The University of Manchester, Manchester, Manchester, UK; 10 Henry M. Goldman School of Dental Medicine, Boston University, Boston, Massachusetts, USA; 11 School of Health Sciences, Division of Psychology and Mental Health, Manchester Centre for Health Psychology and Manchester Academic Health Science Centre, The University of Manchester, Manchester, Manchester, UK; 12 Research User Group, Primary Care Centre Versus Arthritis, School of Medicine, Keele University, Keele, Staffordshire, UK

**Keywords:** rheumatology, primary care, musculoskeletal disorders

## Abstract

**Background:**

Brace effectiveness for knee osteoarthritis (OA) remains unclear and international guidelines offer conflicting recommendations. Our trial will determine the clinical and cost-effectiveness of adding knee bracing (matched to patients’ clinical and radiographic presentation and with adherence support) to a package of advice, written information and exercise instruction delivered by physiotherapists.

**Methods and analysis:**

A multicentre, pragmatic, two-parallel group, single-blind, superiority, randomised controlled trial with internal pilot and nested qualitative study. 434 eligible participants with symptomatic knee OA identified from general practice, physiotherapy referrals and self-referral will be randomised 1:1 to advice, written information and exercise instruction and knee brace versus advice, written information and exercise instruction alone. The primary analysis will be intention-to-treat comparing treatment arms on the primary outcome (Knee Osteoarthritis Outcomes Score (KOOS)-5) (composite knee score) at the primary endpoint (6 months) adjusted for prespecified covariates. Secondary analysis of KOOS subscales (pain, other symptoms, activities of daily living, function in sport and recreation, knee-related quality of life), self-reported pain, instability (buckling), treatment response, physical activity, social participation, self-efficacy and treatment acceptability will occur at 3, 6, and 12 months postrandomisation. Analysis of covariance and logistic regression will model continuous and dichotomous outcomes, respectively. Treatment effect estimates will be presented as mean differences or ORs with 95% CIs. Economic evaluation will estimate cost-effectiveness. Semistructured interviews to explore acceptability and experiences of trial interventions will be conducted with participants and physiotherapists delivering interventions.

**Ethics and dissemination:**

North West Preston Research Ethics Committee, the Health Research Authority and Health and Care Research in Wales approved the study (REC Reference: 19/NW/0183; IRAS Reference: 247370). This protocol has been coproduced with stakeholders including patients and public. Findings will be disseminated to patients and a range of stakeholders.

**Trial registration number:**

ISRCTN28555470.

**Strengths and limitations of this study**

- Large, publicly funded trial addressing an important unresolved question around affordable, effective non-pharmacological management of painful knee osteoarthritis.
- Recruitment from a broad range of settings (general practice, physiotherapy services and self-referral following awareness raising).
- The intervention arm of our pragmatic design stratifies the provision of brace type based on predominant clinical and radiographic features at presentation, incorporates adherence support components and is delivered by physiotherapists—a scalable model for future implementation.
- Restricted range of off-the-shelf brace options included.
- The trial is powered for the overall comparison between treatment arms but not for subgroup analyses of different brace types.

## Introduction

Symptomatic knee osteoarthritis (OA) affects an estimated 10% of adults aged over 55 years^1^ and has a significant impact on population health, healthcare demand and societal costs. Trends in disability-adjusted life-years attributed to OA,^2^ the number and rate of primary knee replacements,^3–6^ and rates of new presentations to primary care^7^ all suggest an increasingly common problem that accounts for up to 0.5% gross domestic product in high-income countries.^8 9^

In the UK, the National Institute for Health and Care Excellence (NICE) currently recommends that people with OA who have ‘biomechanical joint pain with or without instability should be considered for assessment for bracing as an adjunct to their core treatments’.^10^ However, there remains a lack of high-quality trials on their clinical and cost-effectiveness. While some international clinical guidance recommend bracing for knee OA,^11 12^ several others have been unable to make a recommendation due to the limited evidence base.^13–16^

A Cochrane review, originally published in 2005^17^ and updated in 2015,^18^ identified five randomised controlled trials (RCTs),^19–23^ with sample sizes ranging from 33 to 117, that compared a type of brace for OA of the knee versus no treatment or other treatment such as restricted activity, patient education, exercise, pharmacological treatment and orthoses or surgical treatment. The review concluded that ‘low-quality evidence suggests that people with OA who use a knee brace may have little or no reduction in pain or improvements in knee function and quality of life’. Since then, five further systematic reviews^16 24–27^ and one narrative review^28^ have been published and identified a total of 13 RCTs,^29–41^ (two RCTs were published after the Cochrane review).^40 41^ These concluded that either the existing evidence is insufficient,^16 26 27^ or is consistent with small-to-moderate effects on pain and function for unloader braces^24^ and soft sleeve braces^25^; therefore, further investigation is warranted. Only three trials^20 21 29^ and one observational study^42^ followed participants up for 6 months or longer.

The current trial was designed in response to a commissioned call from the National Institute for Health Research Health Technology Assessment programme for an efficient and pragmatic trial to investigate, in primary care, the clinical and cost-effectiveness of knee braces in the management of knee OA.

## Objectives

The overall aim of the ‘PROvision of braces for Patients with knee OA’ (PROP OA) trial is to determine the clinical and cost-effectiveness of adding knee bracing (matched to patients’ clinical and radiographic presentation and with adherence support) to advice, written information and exercise instruction compared with advice, written information and exercise instruction alone, in adults with symptomatic knee OA.

Specifically, our primary objective is to determine, in adults with symptomatic knee OA, if the advice, written information and exercise instruction plus knee brace is superior to advice, written information and exercise instruction alone for Knee Osteoarthritis Outcomes Score (KOOS)-5, a composite score of patient-reported pain, other symptoms, activities of daily living (ADLs), function in sport and recreation and knee-related quality of life, at 6 months.

Key secondary objectives are to determine, in adults with symptomatic knee OA:

- If the advice, written information and exercise instruction plus knee brace is superior to advice, written information and exercise instruction alone for KOOS-5 at 3 and 12 months.
- If the advice, written information and exercise instruction plus knee brace is superior to advice, written information and exercise instruction alone for the separate components of KOOS-5 (patient-reported pain, other symptoms, ADLs, function in sport and recreation and knee-related quality of life) at 3, 6 and 12 months.
- Cost-effectiveness of the advice, written information and exercise instruction plus knee brace compared with advice, written information and exercise instruction alone.

Other secondary objectives are to determine:

- If the advice, written information and exercise instruction plus knee brace is superior to advice, written information and exercise instruction alone for: self-reported pain; instability (buckling); treatment response; physical activity; social participation; arthritis self-efficacy.
- Safety of knee bracing in adults with symptomatic knee OA ((serious)- related adverse events (SAE)).
- Acceptability and experiences of the trial procedures and interventions to participants receiving and physiotherapists delivering, the trial interventions.
- Adherence to interventions, including exploring barriers and enablers of adherence to brace use in participants allocated to the advice, written information and exercise instruction plus knee brace intervention.

A priori exploratory subgroup analyses will explore the effectiveness of the knee brace plus advice, written information and exercise instruction intervention versus advice, written information and exercise instruction alone by: (1) predominant knee compartmental involvement, (2) knee buckling, (3) adherence to brace use, (4) anxiety/depression. We will also explore how often physiotherapists’ clinical judgement on appropriate brace type is changed by plain X-ray findings.

## Design

A multi-centre, primary care, randomised (1:1), parallel-group, superiority trial with internal pilot (see online supplemental datafile 1 for further details relating to the internal pilot and progression criteria). The trial protocol has been coproduced with stakeholders including patients.

10.1136/bmjopen-2020-048196.supp1 Supplementary data

### Setting

Participants will be recruited from National Health Service (NHS) general practice, physiotherapy services and from self-referral within the community following an awareness raising campaign. Interventions will be delivered within PROP OA knee pain clinics based in physiotherapy services at four NHS sites with onsite radiography departments in England (Cheshire, Manchester, Northumbria and Staffordshire).

### Eligibility criteria

The target population is adults aged ≥45 years with symptomatic knee OA, moderate-to-severe pain during weight-bearing activity, with or without knee buckling, who have no current or recent knee brace use but who would be willing to consider using a knee brace (table 1).

**Table 1:** Eligibility criteria

Inclusion criteria	Exclusion criteria
Aged 45 years and over Residing in England Clinically significant knee pain on weight bearing (NRS ≥4) With or without knee instability (buckling) Able to have knee X-ray Able to read and write English Access to a mobile phone that can receive SMS text messages Able to give full informed consent Willing to participate	Red flags in the history or clinical examination that may indicate further investigation or referral for possible serious underlying pathology (NICE V.5.1.1.^10^) Vulnerable individuals (eg, in palliative phase of care for cancer, unstable mental health disorders). Inflammatory/crystal arthritis (eg, rheumatoid arthritis, gout, psoriatic arthritis). Significant neurological disorder (eg, stroke, Parkinson’s disease, multiple sclerosis, dementia). Fibromyalgia. Symptoms not attributable to knee OA. Previous major surgery in the knee to be treated (partial/total knee replacement; high tibial osteotomy, not other previous arthroscopic surgery). Autologous cartilage implantation in last 12 months in the knee to be treated On the waiting list for total hip or knee replacement within the next 6 months. Unwilling to wear a knee brace. Brace size unavailable for leg circumference. Knee brace contraindicated (superficial wounds where the knee brace would reside, psoriasis, eczema or poor circulation, arterial insufficiency, or severe varicosities that could result in skin at risk with regular brace wear, a history of thrombophlebitis in either leg). Significant fixed flexion deformity that prevents fitting of brace. Injection in the knee to be treated within the last 3 months. Recent/routine knee brace wear within the last 3 months. Nursing home resident. Unable to attend clinic. Close family member already a trial participant. Course of physiotherapy for the knee to be treated in the last 3 months.

NICE, National Institute for Health and Care Excellence; NRS, Numerical Rating Scale; OA, osteoarthritis.

### Participant identification

Three methods will be used to identify potential participants, informed by our previous pragmatic trials of treatments for musculoskeletal pain conditions in primary care^43–45^:

#### Identification of general practice consulters

Electronic records of participating general practices will be screened to identify adults aged 45 years and over who have consulted for knee pain in the last 24 months, using knee pain related diagnostic or symptomatic Read/Systematized Nomenclature of Medicine (SNOMED) codes. General practice staff will also identify potentially suitable participants during prospective consultations. In the event of slower than expected recruitment, we will increase the number of participating general practices and increase the time period for previous consultation with knee pain from 24 to 36 months.

#### Screen of physiotherapy referrals

Physiotherapy referrals will be screened to identify adults aged 45 years and over with knee pain potentially due to OA.

#### Self-referral from the community

Not all individuals with knee OA will consult healthcare about their knee problem. Information about the trial will be disseminated in the local communities around the participating sites via an awareness raising campaign. Interested participants will telephone the trial administrator to register their interest.

### Screening

Identified individuals will be mailed a covering letter and Participant Information Leaflet (PIL). If interested in participating they will be asked to telephone the trial team: a trial administrator will check initial eligibility and answer any questions. Individuals who fulfil telephone eligibility criteria and are willing to participate will be invited to attend further face-to-face screening (Clinical Eligibility Assessment). At this visit activities will include:

#### Confirmation of eligibility assessment by a PROP OA-trained physiotherapist

A clinical examination will be undertaken to confirm eligibility and to determine, on clinical grounds, the predominant knee compartment affected (medial tibiofemoral, lateral tibiofemoral, patellofemoral, no clear predominant compartment).

#### Plain X-rays of knees (if needed)

To minimise unnecessary radiation exposure, we will attempt to access and read any knee X-rays taken within 24 months where they exist. We will obtain new knee X-rays for eligible participants who have no knee X-rays within 24 months, or whose knee X-rays are unobtainable/unsuitable (eg, poor-quality images, lack necessary views). All images will be taken in the radiology departments at each site using National Health Service (NHS) standard protocols. The treating physiotherapist will assess the X-rays for the purpose of producing an overall judgement on the most severely affected compartment.

After 2 weeks (to allow reporting of X-rays), eligible participants willing to participate in the trial will attend a ‘Treatment Visit’ where informed consent will be obtained (by a trial physiotherapist), baseline data collected, randomisation performed and trial treatments provided.

### Allocation

Using Keele Clinical Trial Units (CTUs) computerised web-based randomisation service and random number generator, eligible participants recruited to the trial will be randomly assigned to receive either advice, written information and exercise instruction alone or advice, written information, and exercise instruction plus knee brace, using a 1:1 allocation ratio and random permuted blocks. This is a secure randomisation system with emergency telephone back-up. Randomisation will be stratified by physiotherapy clinic site (Cheshire, Manchester, Northumbria and Staffordshire), predominant compartmental distribution of knee OA based on combination of clinical assessment and X-rays, and by presence of instability (buckling). Randomisation will be executed in real time within the Treatment Visit by a clinic administrator. The randomisation code will be allocated only after the participant has been recruited into the trial, and after all baseline data are collected. The randomisation schedule will be password protected to ensure that allocation remains concealed from all staff involved in the randomisation process. Thus our procedures ensure baseline data are collected prior to randomisation, that the allocation is concealed until after the participant has been recruited into the trial and until the moment of randomisation and that the person assigning participants to intervention arm (clinic administrator) has no involvement in the eligibility screen, consent or treatment processes.

### Blinding

Within this trial it is impossible to blind participants or physiotherapists to treatment allocation. However, a trial administrator will oversee collection of baseline questionnaire data ahead of treatment allocation and a trial administrator will remain blind to treatment allocation to enable them to conduct Minimum Data Collection (MDC) over the telephone free of allocation bias. The trial statistician who will be performing analysis of the data collected will also be blind to treatment allocation.

### Interventions

#### Comparator: advice, written information and exercise instruction

A single, face-to-face, 20 min consultation with a physiotherapist that, in line with NICE core treatment recommendations for OA,^10^ will include: verbal and written education regarding pathogenesis and prognosis of knee OA and the benefits of exercise, increasing physical activity and weight loss; simple self-help advice on pain management, including home-use of heat/cold, pacing of activities and simple analgesia (we will use the Osteoarthritis Guidebook (https://www.keele.ac.uk/media/keeleuniversity/ri/primarycare/pdfs/OA_Guidebook.pdf) with minor trial-specific changes adapted by Public and Patient Involvement and Engagement (PPIE)); and advice to complete a home-based lower limb exercise programme focusing on muscle strengthening, knee range of movement and proprioception (see online supplemental datafile 2). This reflects ‘best care’ routinely available in primary care where knee OA is typically managed. The content of the advice and exercises was informed by our previous knee OA trials testing physiotherapist led OA care.^44 46^

10.1136/bmjopen-2020-048196.supp2 Supplementary data

#### Intervention: advice, written information and exercise instruction plus knee brace with adherence enhancing component

An initial 1-hour face-to-face treatment session with a physiotherapist, a 30 min face-to-face follow-up appointment with the physiotherapist 2 weeks later, and motivational prompts to enhance brace adherence sent via SMS text message over 6 months.

#### Initial treatment session

Participants will receive advice, written information and exercise instruction as described above, plus knee brace and adherence enhancing components.

##### Knee brace

In addition to advice, written information and exercise instruction, participants will be given either a patellofemoral, tibiofemoral unloading, or neutral stabilising knee brace according to whether their pattern of knee OA was predominantly patellofemoral OA, tibiofemoral OA (medial/lateral) or generalised knee OA. This will be based on clinical assessment and X-ray findings, but also taking into account current and desired level of physical activity, ability to don/doff brace, willingness to wear the brace type, and immediate symptom response when the brace is tried on and tested in clinic. Braces will be fitted to ensure maximum comfort (eg, hinges contoured, straps adjusted and cut to match participants’ body shape and size). Dose of brace use will be individually tailored. Participants will be advised to wear the brace on painful weight-bearing activity, with a starting minimum usage of 1 hour on ≥2 days/week, gradually increased based on tolerance to wearing the brace on all painful weight-bearing activities up to a maximum of 8–12 hours/day. Individuals will be advised to wear the brace for 6 months and continue to wear it beyond this time if they find it beneficial. Verbal and written information will be provided on brace application and care, including cleaning instructions and what to do in instances of slippage, discomfort or skin irritation. Supporting patient material (eg, written information, short video clips) on brace application produced by the brace manufacturers will be available.

The braces selected for use in this trial are from the two most-referenced manufacturers in the medical literature (Össur and Donjoy)^47^ and the manufacturer of the patellofemoral brace (Bioskin) previously demonstrated to be clinically effective in a similar population as intended with the current trial.^40^ The braces, by type, are: patellofemoral—Bioskin Q Brace; tibiofemoral unloading—Össur Unloader One (first choice), Donjoy OA Nano (second choice); neutral stabilising—Össur Formfit Knee Hinged. These have been selected to provide an appropriate brace within each brace type, based on previous trial experience and evidence, PPIE feedback and expert opinion (including Clinical Advisory Group (CAG) members’ views).

##### Brace adherence enhancing component

Brief motivational interviewing (MI) techniques will be used to build participants’ intrinsic motivation and resolve ambivalence about adhering to brace use.^48^ The techniques will be based on brief strategies to enhance motivation to change,^48^ including both communication strategies and motivational techniques. Communication strategies include: (1) open-ended questions that invite patients to provide their thoughts about brace adherence in a non-judgemental atmosphere, (2) use of affirmations or statements that creates rapport and recognises participants’ strengths and small steps towards improved adherence; (3) reflective listening to convey provider understanding, provide new perspectives on their situation and help providers deal with resistance and (4) use of summaries to help resolve ambivalence and highlight the patient’s self-motivational statements. Motivational techniques include (1) helping the patient weigh the costs and benefits of adherence to brace use, (2) providing education and feedback on adherence using the ‘elicit-provide-elicit’ process, (3) exploring barriers and facilitators of motivation to adhere and confidence to adhere, and (4) discuss how adherence can enhance, rather than detract from, things they most highly value (values clarification). Participants will be provided with a diary which they can use to record whether they have worn the brace, for how long, if not what were the barriers to wearing the brace, and what are the possible solutions to those barriers.

#### Follow-up treatment session

During the follow-up consultation the physiotherapist will check response to, and fit of the brace. For participants who report tolerating it well and finding it helpful, brace use will be increased for longer durations of painful weight bearing activity. If participants are experiencing discomfort or not finding it helpful, brace fit and dose may be reduced. In extreme cases, if the brace is not tolerated, it may be changed. Adherence to brace use will be reviewed and addressed using brief MI techniques and based on information provided within the brace diary.

#### Motivational prompts to enhance brace adherence

Motivational prompts to encourage brace adherence will be sent to individuals receiving advice, written information and exercise instruction plus knee brace intervention via Short Message Service (SMS) text message. The content of these texts will be tailored to the level of brace use reported by the participant (low: wearing knee brace on less than 2 days per week; mid: wearing knee brace on 2–4 days per week for 1 or more hours per day or wearing knee brace on 5–7 days per week for 1–3 hours per day: high: wearing knee brace on 5–7 days per week for 4 or more hours per day). For example, patients who have low use will receive texts that target their ambivalence about wearing the brace; those with moderate levels of use will receive texts about problem solving and barriers along with some motivational strategies and those with the highest level of use will receive congratulatory messages and messages regarding potential benefits they may have incurred. Motivational prompts will be sent weekly for the first 4 weeks, fortnightly for the next 8 weeks, and then monthly until the intervention period ends at 6 months.

### Cointerventions

All participants will be advised that they can continue to access usual healthcare, including medications and consultations with other health professionals. Participants allocated to the comparator arm will be asked not to wear a knee brace for the 6-month period following randomisation. Details of cointerventions will be recorded in follow-up questionnaires.

### Study training

Physiotherapists delivering trial interventions will be representative of the range of physiotherapists that patients would see beyond the trial in the UK NHS setting. Participating physiotherapists will deliver both interventions and will receive a 3-day PROP OA training programme prior to the trial starting, regular reminders regarding the content of treatment sessions for the first 3 months, and refresher training at 6 months and 1 year. Training will cover all trial aspects including: clinical assessment; reading and interpretation of plain knee X-rays to judge compartmental involvement and inform brace allocation; provision of trial interventions, including dovetailing the provision of knee braces with brief MI techniques to facilitate brace adherence and Good Clinical Practice (GCP) and trial procedures. To promote protocol fidelity, the training programme will be delivered using interactive group discussion, problem solving, case examples and role play. In addition, physiotherapists will receive a PROP OA manual to following when delivering both interventions. Physiotherapists will record intervention provision using case report forms (CRFs), which will be monitored over the course of the trial and used to assess ongoing treatment fidelity.

### Outcomes

The end points are defined as: primary end point at 6 months for clinical effectiveness and at 12 months for cost-effectiveness analysis, and withdrawal for any reason. A schedule of enrolment, interventions and assessments is provided in table 2.

**Table 2:** Schedule of enrolment, interventions and assessments

Time point	Enrolment	Random allocation	Post-randomisation			
	−8 to −0 weeks	0	2 weeks	3 months	6 months	12 months
**Enrolment**						
Telephone eligibility assessment	X					
Informed consent to assessment	X					
Clinical eligibility assessment	X					
Knee X-ray acquisition/reporting	X					
Knee X-ray assessment		X				
Informed consent to randomisation, treatment		X				
Random allocation		X				
**Interventions**						
Advice, written information and exercise instruction		X				
Advice, written information and exercise instruction+knee brace		X	X			
Assessment**						
Demographics	X					
Medical history and physical assessment	X					
Pain manikin	X					
Frequent knee symptoms in last month	X					
KOOS-5*	X			X	X	X
KOOS Activities of Daily Living†	X			X	X	X
KOOS Pain†	X			X	X	X
KOOS Symptoms†	X			X	X	X
KOOS Sports/Recreation†	X			X	X	X
KOOS Quality of Life†	X			X	X	X
KOOS-4	X			X	X	X
Knee pain on weight-bearing activity (0–100 NRS)†	X			X	X	X
Intermittent and Constant Pain (ICOAP)	X			X	X	X
Knee buckling‡	X			X	X	X
Physical activity (IPAQ-E)	X			X	X	X
Arthritis self-efficacy	X			X	X	X
HADS: Anxiety	X					
HADS: Depression	X					
PROMIS Social participation	X			X	X	X
Adverse events			X	X	X	X
Adherence§			X	X	X	X
Patient global rating of change¶				X	X	X
OARSI-OMERACT responder criteria				X	X	X
Treatment acceptability				X		
EuroQol EQ-5D-5L	X				X	X
Healthcare resource use (NHS/private)	X			X	X	X
Out-of-pocket expenses	X				X	X
Time off work	X				X	X

*Primary outcome.

†Key secondary outcomes.

‡Single item used for stratified randomisation, multiple items used for outcome evaluation.

§Obtained in part through: two-way SMS text messages at weeks 1, 2, 3, 4, 6, 8, 10, 12, 16, 20, 24, 52; self-report via questionnaire at 3, 6 and 12 months.

¶Measure used only to classify OMERACT-OARSI responder.

**Close-out at 12 months for analysis of clinical effectiveness.

HADS, Hospital Anxiety and Depression Scale; ICOAP, Intermittent & Constant Osteoarthritis Pain; IPAQ-E, International Physical Activity Questionnaire-Elderly; KOOS, Knee Osteoarthritis Outcomes Score; NHS, National Health Service; NRS, Numerical Rating Scale; OARSI-OMERACT, Osteoarthritis Research Society International; PROMIS, Patient-Reported Outcomes Measurement Information System.

The primary outcome is patient-reported composite knee score of patient reported pain, other symptoms, ADLs, function in sport and recreation and knee-related quality of life (KOOS-5).^49^

#### Secondary endpoints/outcomes

Secondary outcomes include patient reported pain, other symptoms, ADLs, function in sport/recreation and knee-related quality of life (KOOS subscales),^49^ pain (pain on weight-bearing activity (Numerical Rating Scale)), intermittent and constant pain (ICOAP)^50^; instability (buckling)^51^; Osteoarthritis Research Society International responder criteria^52 53^; physical activity (International Physical Activity Questionnaire-Elderly^54^; social participation (PROMIS)^55 56^; arthritis self-efficacy^57^; treatment acceptability^58^; (SAE). KOOS Pain items contain those needed to score Western Ontario and McMaster Universities Osteoarthritis Index (WOMAC) Pain, KOOS ADLs score is the same as WOMAC Physical Function score and KOOS-4 can be easily computed: all of which permits wider comparison of findings and facilitates future individual participant data (IPD) meta-analysis (see table 2).

#### Cost-effectiveness

Outcomes include the EQ-5D-5L questionnaire^59^ and self-reported knee OA-related resource use related to both primary and secondary care consultations, inpatient stays and treatment including surgery. Data will be collected within the trial to determine the cost of the interventions (physiotherapist time, cost of braces). Unit costs from standard UK sources will be sought for all healthcare resource use items. Data on broader costs will also be collected, related to both out of pocket costs (eg, over-the-counter medications), private healthcare and time off work to calculate productivity losses.

#### Time to and receipt of knee surgery (knee arthroscopy and knee joint replacement)

All participants will be invited to consent to linkage of their trial data to the Hospital Episode Statistics and National Joint Registry and medical record review to enable future evaluation of receipt of knee arthroscopy and knee joint replacement.

#### Adherence

Adherence to interventions will be measured via self-report in follow-up questionnaires (using similar questions to those we have used in previous trials of non-pharmacological care for OA).^44^ In those randomised to advice, written information and exercise instruction plus knee brace, SMS text messages will also seek data on number of days per week and hours per day of brace usage, sent on a tapering schedule over the first 6 months of follow-up, with a text message also at 12 months. We will seek to include an objective measure of adherence to brace use, with participants blinded to this.

#### Adverse events

The occurrence of adverse events considered to be related to the trial interventions for each intervention will be monitored and assessed using CRFs, contact with the trial team, physiotherapist report and follow-up questionnaires. Expected adverse events from knee braces for knee OA include: swelling, blisters and skin irritation. An expected adverse event from unaccustomed exercise and physical activity is temporary, mild muscle soreness. An SAE is an untoward event that: (1) results in death; (2) is life threatening; (3) requires hospitalisation or prolongation of existing hospitalisation; (4) results in persistent or significant disability/incapacity; (5) consists of a congenital anomaly/birth defect or (6) is otherwise considered medically significant. Where an SAE is considered to be potentially related to trial procedures, reporting procedures will be followed that are in accordance with GCP guidance. All SAEs either confirmed or suspected to be related to the trial procedures will be considered by the external monitoring committees.

### Data collection

**Figure 1:**
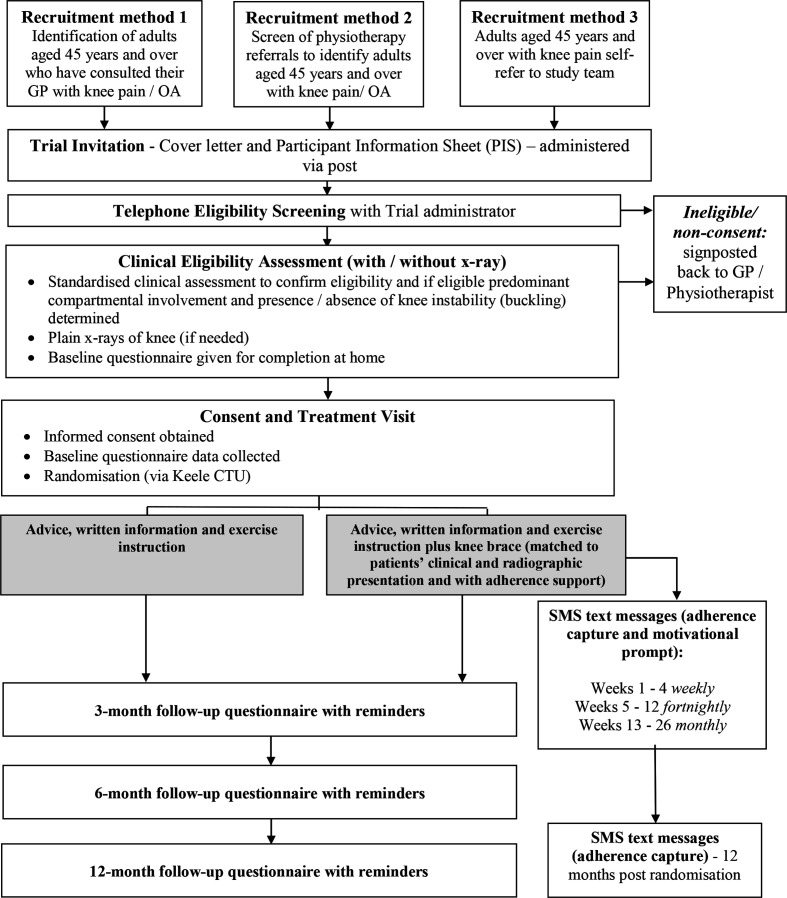
Participant flow and timeline. CTU, Clinical Trial Unit; GP, general practitioner; OA, osteoarthritis.

All trial participants will be asked to complete a paper questionnaire at, or just prior to, the baseline clinic appointment and a posted questionnaire after 3, 6 and 12 months postrandomisation (see table 2 for a summary of questionnaire content). Standard Keele CTU procedures will be followed to maximise follow-up, including postcard and questionnaire reminders. In addition, a £10 gift voucher will be sent to participants along with their 3, 6 and 12 months follow-up questionnaires.^60^ At 6 months follow-up, the primary end point, non-responders will be approached for MDC 2 weeks after the reminder questionnaire is mailed. MDC is a shorter version of the self-report outcome questionnaire and will be used to collect the primary and limited secondary outcome measures (KOOS-5), global change scores and EQ-5D-5L, along with date of birth and gender to ensure the data are provided by the intended participant. If no response to the MDC questionnaire, we will attempt to collect minimum data over the telephone by a trial administrator. Data on self-reported adherence to brace use (in the advice, written information and exercise instruction plus knee brace intervention group) will be collected by two-way SMS text messages at 1, 2, 3, 4, 6, 8, 10, 12, 16, 20, 24 and 52 weeks. A schematic diagram of the participant flow chart is provided in figure 1.

Bracing trials show standardised effect sizes (ES) for short-term improvements in knee pain and function of 0.33–0.56 and 0.22–0.48, respectively, for tibiofemoral unloading braces^24^ and 0.61 and 0.39, respectively, for soft neoprene sleeve braces.^25^ Our trial is powered to detect a between-group ES of 0.35 (small-to-medium effect) in the primary outcome at 6 months with two-sided 5% significance and 90% power, which, assuming an SD of 23 as estimated from previous trial data,^44^ equates to a minimum clinically important difference (MCID) of 8-points on the KOOS-5; an MCID value that aligns with published evidence for the tool.^61^ We will randomise 434 patients, recruited over a period of 24 months, to allow for 20% lost to follow-up at 6 months,^44 46 62^ (target n at 6 months=346; 173/arm). We have not inflated our sample size for therapist effects as each physiotherapist will be trained to deliver both interventions, however, the therapist will be included as a covariate in a sensitivity analysis of the treatment models to increase model power.^63^

### Statistical methods

A separate a priori data analysis plan will be written to describe all trial analysis. Consequently, only a brief outline is outlined below.

A Consolidated Standards of Reporting Trials flow diagram will describe the flow of participants through the study and will include reasons for trial withdrawal if given. Descriptive statistics will be used to describe the key baseline characteristics of participants at each stage of recruitment and follow-up, and by intervention arm. The primary outcome analysis will be on an intention-to-treat basis and will compare the primary outcome (KOOS-5) at the primary endpoint (6 month follow-up) for advice, written information, and exercise instruction plus knee bracing versus advice, written information and exercise instruction alone. Secondary analysis will include the analysis of the primary outcome at 3 and 12 months (ie, the secondary endpoints) and the secondary clinical effectiveness outcomes at 3, 6 and 12 months. Analysis of covariance will be used to model outcomes that are continuous and logistic regression for those that are dichotomous. All models will be reported after adjustment for a priori analysis covariates (as defined in the a priori analysis plan) and after imputation of missing data. Treatment effect estimates will be presented as mean differences or ORs (as appropriate) with 95% CIs. Longitudinal mixed-effects models will also be used to evaluate the treatment effect at each single time point and over time to check that results are consistent with those derived from the primary analysis.

Exploratory subgroup analyses will be performed (if numbers in each subgroup are sufficient for these analyses to be feasible) based on the presence of knee instability (buckling), predominant knee compartment involved, level of adherence and anxiety/depression. Participants will also be included in a per-protocol analysis if they meet prespecified inclusion criteria, defined fully in the a priori analysis plan. Cross-tabulation and the kappa statistic will be used to test how accurate brace allocation would have been if based on clinical judgement alone, rather than on clinical assessment findings and X-ray assessment combined. Treatment acceptability will be described using numbers and percentages. Any adverse events and protocol violations will be reported throughout the trial by intervention arm. No interim analysis will be undertaken during the trial to assess the clinical effectiveness of advice, written information and exercise instruction plus knee bracing over advice, written information and exercise instruction alone, however, an internal pilot trial phase will be conducted with prespecified progression criteria (online supplemental datafile 1).

### Economic evaluation

#### Within trial economic evaluation

An economic evaluation will be undertaken alongside the trial to estimate the cost-effectiveness of advice, written information and exercise instruction plus knee bracing versus advice, written information and exercise instruction alone in the management of knee OA. An incremental cost–utility analysis will estimate cost per quality-adjusted life year (QALY) over 12 months follow-up. QALYs will be calculated from responses to the EQ-5D-5L questionnaire using the ‘area under the curve’ approach. The cross-walk value set to obtain utility scores, in line with current NICE recommendations^64^ and the more recent English value set will be used in a sensitivity analysis. The base-case analysis will be from a healthcare perspective, with an additional analysis from a societal perspective taking into account out-of-pocket costs and work productivity losses. Mean resource use (for each category of healthcare usage) and mean total costs will be calculated for all trial participants. Analysis of productivity losses will use the human capital approach, and the self-reported days of absence will be multiplied by the respondent-specific wage rate. Multiple imputation will be used to impute all missing values for the EQ-5D and total cost estimates for missing data at follow-up. Incremental cost–utility analysis will be undertaken to estimate the incremental cost per QALY gained, adjusting for baseline covariates. The robustness of the results will be explored using sensitivity analysis. Cost-effectiveness acceptability curves will also be produced to reflect the probability the intervention will be cost-effective at different cost per QALY willingness to pay thresholds.

#### Model-based health economic analysis

Decision modelling, using a Markov model, will also be undertaken to extend beyond 12 months follow-up, to extrapolate costs and QALYs over a lifetime time horizon. The cost–utility analysis will be from an NHS perspective, with discounting of costs and outcomes at 3.5%. In addition to trial data, the model will be populated with data from existing literature on the natural history of symptomatic knee OA, risks associated with surgery and quality of life, and national data on all-cause mortality. The model will be subject to deterministic sensitivity analysis by changing individual parameter values and changing model assumptions, and probabilistic sensitivity analysis to simultaneously incorporate all parameter uncertainty. Cost-effectiveness planes and cost-effectiveness acceptability curves will be presented to show the probability that advice, written information and exercise instruction plus knee bracing is cost-effective at different cost/QALY thresholds.

### Nested qualitative studies

In addition to qualitative interviews undertaken in the internal pilot phase (online supplemental datafile 1), a theoretically informed qualitative study will be completed within the main trial, which builds on our previous work exploring acceptability of, and adherence to, physiotherapy-led interventions for individuals with knee OA, and barriers and facilitators to non-pharmacological interventions (exercise and physical activity) in this population.^65–67^

To help explain the results of the trial in terms of the comparable clinical effectiveness of interventions, a qualitative study will investigate contextual factors, and barriers and enablers to brace use in individuals with knee OA. Drawing on normalisation process theory (NPT)^68 69^ and the theoretical domains framework (TDF),^70^ we will investigate patient and physiotherapy perspectives and will undertake:

Semistructured interviews (face to face or telephone) with ≤40 participants postintervention (at 6 months follow-up) (n≤20 from each treatment arm). Participants will be purposefully sampled from both treatment arms (using data from trial questionnaires and SMS text messages) to ensure a diverse range of characteristics including age, sex, knee compartmental involvement, baseline pain severity, presence/absence of buckling, perceived overall improvement and level of adherence. The interview topic guide will explore participants’ experiences of trial interventions, adherence to interventions (including barriers and enablers to brace adherence in the advice, written information and exercise instruction plus knee brace intervention arm), and impact of interventions on participants’ symptoms, functioning and quality of life. Perceived harms and adverse events from interventions will also be explored. Data collection and analysis will be carried out iteratively so that emerging themes can be effectively explored in subsequent interviews.^71^ Sampling will continue until no new themes emerge.

Semistructured interviews will be conducted with physiotherapists who delivered trial interventions (n≤16) after they have treated their final trial participant. Interviews will provide insight into experiences of implementing, embedding, accepting and integrating both advice, written information and exercise instruction and the addition of knee bracing into NHS physiotherapy services. The topic guide will cover the appropriateness of the trial training programme, physiotherapists’ views of their roles in the trial in terms of delivering usual practice (advice/education/exercise prescription) but in only one treatment session, and in the advice, written information and exercise instruction plus knee bracing arm, the extra activity including using a standardised clinical assessment to identify compartment of knee affected by OA, assessment of X-rays, prescribing a brace and supporting the patient to use it, changing it where necessary, and using brief MI techniques specifically to facilitate adherence to brace use.

All interviews will be audiorecorded, transcribed verbatim, checked and anonymised for analyses. Initially, each interview transcript will be read and reread to identify and code discrete parts of the data that represent a particular concept. As analysis continues, using principles of constant comparison,^71^ data will be closely examined for similarities and differences, and groups of words or phrases representing the same concept will be grouped into themes. Emerging codes and themes will be discussed and agreed with members of the study team (including PPIE team members) on an ongoing basis and applied to the dataset with ongoing refinement as needed. Inductive analysis (described above) will precede deductive analysis and mapping of themes to TDF or NPT constructs.^68 70^ This layered approach enables a rich interpretative analysis to be completed as emergent issues are identified ahead of making sense of data according to theoretical constructs.^68 72^ Data will be used to inform an implementation strategy in conjunction with Keele’s Impact Accelerator Unit.

## Data management

Self-report questionnaires, SMS text messages and CRFs will form the basis of data collection. All data collected during the course of the trial will be handled and stored in line with Keele University’s Data Security procedures and Standard Operating Procedures (SOPs), which are in accordance with the Data Protection Act 2018, other relevant regulations and GCP guidelines. If a participant withdraws consent from trial intervention and/or further collection of data, their data will remain on file and included in the final analysis. At the end of the trial, data will be securely archived in line with the Sponsor’s procedures for a minimum of 5 years. Data held by Keele CTU will be archived in the designated Keele CTU archive facility and site data and documents will be archived at the participating sites. Following authorisation from the Sponsor, arrangements for confidential destruction will then be made.

## Trial organisation

The trial steering committee (TSC) met prior to ethics application in order to agree the final protocol, and will continue to meet at agreed time intervals over the course of the trial. An independent data monitoring committee approved the protocol and reviews the safety of the trial. Detailed reports focusing on interim safety, recruitment and retention are prepared by Keele CTU at approximately 6 monthly intervals. All data collection, database design, data input and cleaning, as well as trial oversight procedures, are in line with the Keele University SOPs and the conditions of the grant. Data will be centrally monitored for quality and completeness by Keele CTU.

## Public and patient involvement

Two patient representatives are members of the trial team and helped write the grant application and actively contributed to revising the trial protocol following CAG and Panel feedback. A 3-hour workshop was convened with five patient representatives with knee OA to discuss recruitment, participant flow, interventions (including trying on/ discussing patellofemoral, tibiofemoral unloading and neutral stabilising knee braces), outcome measures and dissemination. Comments about the trial were obtained from the general public via the interactive VoiceNorth discussion forum and specific feedback from nine VoiceNorth members (http://www.voicenorth.org/). As a result, changes were made to the recruitment strategy, outcome measures and the collection of information on reasons for non-adherence.

To further refine the design of the trial, a second PPIE workshop was convened in the pretrial phase to finalise the adherence-enhancing SMS intervention (eg, content of text messages). To help inform the development of the PROP OA trial protocol, we also formed, and convened two workshops with, a CAG consisting of multidisciplinary clinicians involved in the PROP OA. Our PPIE coapplicants actively participated in these workshops which shaped the eligibility criteria, intervention content and processes of brace selection.

Internal pilot phase findings and potential changes needed to the trial will be discussed with lay members. To ensure ongoing oversight PPIE study members will attend TMG meetings, and assist in the interpretation of qualitative findings. Two independent lay members will also sit on the TSC. Finally, PPIE study members will play an important role in developing easily understandable key messages about trial findings for our dissemination strategy. With the CAG, PPIE members will inform the implementation strategy with the Impact Accelerator Unit. We have an established track record in publishing our PPIE related work.^43–78^ All PPIE activity will follow our written framework for PPIE involvement that is based on INVOLVE guidelines,^79^ and will be supported by our User Support Worker.

## Ethics and dissemination

### Ethical and regulatory considerations

This clinical trial has been designed, and will be run, in accordance with the principles of GCP. The trial involves the investigation of interventions in practice. All braces used in the trial are CE marked mass-product (not custom-made) medical devices being used for their intended purpose.

All patients will receive advice, written information and exercise instruction that aligns with best evidence recommendations from NICE.^10^ All interventions are deemed safe, with few and minor expected adverse events (e skin irritation from knee braces, muscle soreness from exercise). Participants in both intervention arms will be able to consult for healthcare in addition to the care they receive within the PROP OA trial, and this will be recorded and analysed. Participation in the trial may involve exposure to ionising radiation in the form of plain radiography to obtain knee X-rays at baseline. Imaging procedures will be compliant with the Ionising Radiation (Medical Exposure) Regulations 2000 and the amendments in 2006 and 2011.

### Research ethics approval

This project received a favourable ethical opinion from North West Preston Research Ethics Committee (IRAS Reference: 247370; REC Reference: 19/NW/0183) and approval from the Health Research Authority (HRA) and Health and Care Research in Wales (HCRW) on (19 June 2019). The imaging protocol was reviewed prior to REC submission by an independent Medical Physics Expert and Clinical Radiation Expert.

### Protocol amendments

This is an edited version of full current protocol V.2.2 (4 March 2020). The full version of the protocol is available at https://fundingawards.nihr.ac.uk/award/16/160/03.

## Dissemination policy

Our main findings on the clinical and cost-effectiveness of adding knee bracing to advice, written information and exercise instruction will have important implications for patients and the NHS. To ensure that the outputs from the research inform clinical practice, our dissemination strategy will use multiple modes of communication to reach five key audiences for this research: patients with knee pain/OA and the wider public; healthcare professionals; Clinical Commissioning Groups and commissioning organisations; external statutory bodies, patient groups and charities; academia. Keele’s Impact Accelerator Unit will work close with the research team throughout the study to shape an implementation strategy and identify key innovations for adoption.

## Availability of data and materials

Keele University is a member of the UK Reproducibility Network and committed to the principles of the UK Concordat on Open Research Data. The School of Medicine and Keele CTU have a long-standing commitment to sharing data from our studies to improve research reproducibility and to maximise benefits for patients, the wider public and the health and care system.

Metadata, including study protocol, statistical analysis plan, data dictionaries and key study documents (PIL, blank/coded CRFs, consent form), will be deposited on a publicly accessible repository. Deidentified IPD that underlie the results from this trial will be securely stored on servers approved by a government-backed cyber security scheme and made available to bona-fide researchers on reasonable request via our controlled access procedures. Unless there are exceptional circumstances, data will be available on publication of main study findings or within 18 months of study completion (whichever is earlier) and with no end date. Data requests and enquiries should be directed to medicine.datasharing@keele.ac.uk. We encourage collaboration with those who collected the data, to recognise and credit their contributions.

The data generated from this trial will remain the responsibility of the Sponsor. Release of data will be subject to a data use agreement between the sponsor and the third party requesting the data. Deidentified IPD will be encrypted on transfer.

## Supplementary Material

Reviewer comments
