# Supplementary material for: Clinical and cost-effectiveness of bracing in symptomatic knee osteoarthritis management: protocol for a multicentre, primary care, randomised, parallel-group, superiority trial
Source: BMJ Open. 2021 Mar 26;11(3):e048196. doi: 10.1136/bmjopen-2020-048196 (PMC8006841; doi:10.1136/bmjopen-2020-048196)
Supplement: Supplementary data [file bmjopen-2020-048196supp001.pdf]

SUPPLEMENTARY DATAFILE 1. INTERNAL PILOT

An internal pilot will be completed to enable us to check our assumptions about the sample size and to test key trial processes and logistical issues including recruitment, intervention fidelity, adherence to brace use, follow-up and retention, and outcome measurement.

Objectives

Specific objectives of the internal pilot are to:

1) Check the numbers of eligible patients and rate of recruitment overall per month, per site per month, and per recruitment method (identification of General Practice consulters, screen of physiotherapy referrals, self-referral in the community following awareness raising)
2) Explore intervention fidelity and participant adherence to brace use
3) Check the trial retention rate

Methods

The internal pilot will last for 9 months, commencing from the start of recruitment (month 7 of the study timeline). All 4 sites will be included within the internal pilot, and the data collection methods used will be as per those of the full trial (as described above, and with an objective measure of brace adherence if deemed feasible within the pre-trial phase).

Data collection

Data from the following sources may be used within the internal pilot phase: telephone eligibility screening case report form; baseline, 3 month follow-up questionnaires (where available); text messaging (brace adherence data - advice, written information, and exercise instruction plus knee brace only); physiotherapist case report forms; recruitment database; follow-up database; adverse event log; preliminary qualitative interviews with participants and physiotherapists.

Internal pilot qualitative study

Based on best practice guidance,[s1] the aims of the qualitative study within the internal pilot are to investigate: a) the acceptability of trial procedures and interventions; b) adherence to interventions among participants (including barriers and enablers to brace use in individuals in the advice, written information, and exercise instruction plus knee brace intervention arm); and c) barriers and enablers to successful delivery of interventions among trial physiotherapists. We will draw on Normalisation Process Theory (NPT)[s2] to investigate the work required to deliver and adhere to bracing in the trial,

and to understand treatment burden.[s3] We will draw on the Theoretical Domains Framework (TDF)[s4] to understand behavioural determinants of participant adherence to, and physiotherapist delivery of, interventions. We will undertake:

- Semi-structured one-to-one interviews with up to 20 trial participants (n≤10 in each intervention arm) at 3-months follow-up
- Semi-structured one-to-one telephone interviews with physiotherapists who have delivered trial interventions within the internal pilot phase (n=up to 10).

The findings will be used to inform the decision to progress to the main trial, identify necessary changes to the trial processes or interventions for the main trial, and to inform on-going physiotherapy training and monitoring within the main trial.

Outcomes

Outcomes of interest for the internal pilot include:

1. Numbers of adults over 45 years with knee pain identified (per site; per recruitment method (including each awareness raising method employed); overall).
2. Number of individuals screened, number and reasons for ineligibility/exclusion/declining participation at telephone and clinical screening stage, and consent/randomisation rates (per site; per recruitment method; overall).
3. Retention and follow-up rates at 3 months (per site; overall).
4. Intervention fidelity measured by number of participants who have received the interventions per protocol, and reasons for any protocol non-adherence (including number of treatment sessions provided, content of treatment sessions, crossover, and off-protocol intervention (including co-interventions sought from participants)) (per site; overall).
5. Brace adherence in those randomised to receive advice, written information, and exercise instruction plus knee brace, measured by self-report via SMS.
6. Relative distribution of most severely affected compartment (both arms) and brace type provided (advice, written information, and exercise instruction plus knee brace intervention arm only).
7. Patient and physiotherapist perceptions of acceptability of trial procedures and interventions.
8. Barriers and enablers to interventions among participants.
9. Barriers and enablers to successful delivery of interventions among trial physiotherapists.

Holden MA, *et al. BMJ Open* 2021; 11:e048196. doi: 10.1136/bmjopen-2020-048196

Sample size

We anticipate that approximately 32 participants in the advice, written information, and exercise instruction plus knee brace intervention arm will have 3 month data in the internal pilot, hence we can estimate the proportion of patients adhering to braces with at least 95% confidence and a 20% margin of error, assuming adherence and fidelity rate of 50% as a worst case scenario for desired precision.[s5] As other important estimates of recruitment and follow-up will be derived from participants with data in both intervention arms, their estimated precision in the internal pilot study will be greater than that for adherence and intervention fidelity (which is estimated from the advice, written information, and exercise instruction plus knee brace intervention arm only).

Progression criteria

A success criteria traffic-light system relating to the internal pilot trial objectives will be used to inform whether we 'stop' 'proceed' or 'proceed but with protocol amendments' to the full trial.[s5] These criteria are shown in the table below and will be finalised with the Trial Steering Committee (TSC) and funder in the pre-trial phase.[s5] We recognise the arbitrary nature of these cut-offs, however having them in place will allow us to identify any issues that are addressable in going forward to a main trial.[s5] Qualitative findings will also be available to the TMG, TSC and funder at the time of decision making regarding progression of the pilot trial. These will be used to help understand the findings of the internal pilot and will be used to help make the final decision as to whether we should stop, proceed, or proceed but with protocol amendments to the full trial.

Progression criteria for internal pilot

	**Proceed to main trial**	**Proceed to main trial with protocol amendments**	**Do not proceed to main trial**
**Recruitment** ***In months 7-9 of recruitment:***	Site has recruited over 4 participants per month; recruited 19 participants per month overall	Site has recruited 3-4 participants per month; recruited 12-18 participants per month overall	Site has recruited fewer than 3 participants per month; recruited fewer than 12 participants per month overall
**Intervention fidelity**	Interventions delivered per protocol for at least 75% of participants (per site; overall)	Interventions delivered per protocol for 45-75% of participants (per site; overall)	Interventions delivered per protocol for fewer than 45% of participants (per site; overall)

Holden MA, *et al. BMJ Open* 2021; 11:e048196. doi: 10.1136/bmjopen-2020-048196

**Adherence to brace use** (advice, written information, and exercise instruction plus knee brace arm only)	At least 75% of participants reporting minimal level of brace adherence at 3 months* (per site; overall)	Between 45-74% of participants reporting minimal level of brace adherence at 3 months* (per site; overall)	Fewer than 45% of participants reporting minimal level of brace adherence at 3 months* (per site; overall)
**Retention and follow-up at 3 months**	At least 75% retention and follow-up at 3 months (per site; overall)	Between 50-74% retention and follow-up at 3 months (per site; overall)	Fewer than 50% retention and follow-up at 3 months (per site; overall)

*Minimal level of brace adherence: wearing the brace for 1 hour on two or more days per week

References

s1. O'Cathain A, Hoddinott P, Lewin S, et al. Maximising the impact of qualitative research in feasibility studies for randomised controlled trials: guidance for researchers. Trials 2015;16:O88.

s2. May CR, Mair F, Finch T, et al. Development of a theory of implementation and integration: Normalization Process Theory. Implement Sci 2009;4:29.

s3. Gallacher K, May CR, Montori VM, Mair FS. Understanding patients' experiences of treatment burden in chronic heart failure using normalization process theory. Ann Fam Med 201;9:235-243.

s4. Michie S, Johnston M, Abraham C, Lawton R, Parker D, Walker A, “Psychological Theory” Group. Making psychological theory useful for implementing evidence based practice: a consensus approach. Qual Safety Health Care 2005;14:26-33.

s5. Avery KN, Williamson PR, Gamble C, et al. Informing efficient randomised controlled trials: exploration of challenges in developing progression criteria for internal pilot studies. BMJ Open. 2017;7:e013537.
