# Supplementary material for: Clinical and cost-effectiveness of bracing in symptomatic knee osteoarthritis management: protocol for a multicentre, primary care, randomised, parallel-group, superiority trial
Source: BMJ Open. 2021 Mar 26;11(3):e048196. doi: 10.1136/bmjopen-2020-048196 (PMC8006841; doi:10.1136/bmjopen-2020-048196)
Supplement: Supplementary data [file bmjopen-2020-048196supp002.pdf]

Knee Exercises

The following exercise programme is designed to increase the movement in your knee joint, strengthen the muscles that support your knee, and improve your balance. Always start any exercise programme very gently. A slight increase in pain is not uncommon during the first 1-2 weeks, since it is normal to feel a generalised ache or fatigue in the muscles you have been exercising. If you experience severe or persistent pain beyond this time point, stop the exercise programme and seek advice from your GP.

How often should I exercise?
Try to do the exercise programme 2 or 3 times per week, waiting for at least 48 hours between sessions.

Try to complete **8-12 repetitions of each exercise. Repeat the same exercise 1-3 times**. Rest for a few minutes before you move onto the next exercise. Build up the number of exercise repetitions you do, and the number of times you complete each exercise gradually.

If you feel a sharp pain whilst you are doing any of the exercises, then this might mean that you are not doing them correctly. Stop and rest until the pain has eased. Read the instructions again, and try again.

How long should I continue for?
It would be beneficial for you and your knee if you are able to continue these exercises and make them a part of your daily routine.

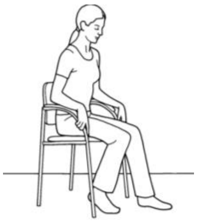

1. Sit on a chair with your feet on the floor.

Bend your knee as much as possible and then straighten it.

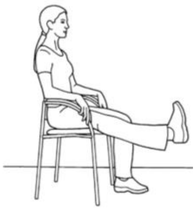

2. Sit on a chair.

Pull your toes up, tighten your thigh muscle and straighten your knee. Hold for approximately 10-30 seconds then slowly relax your leg. Bend your knee as much as possible and then straighten it.

Holden MA, *et al. BMJ Open* 2021; 11:e048196. doi: 10.1136/bmjopen-2020-048196

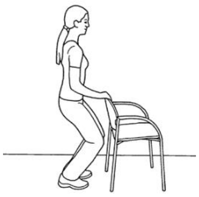

3. Stand in front of a table or chair holding onto it with both hands.

Slowly crouch keeping your back straight and heels on the floor. Stay down for approximately 10-30 seconds and feel the stretching in your buttocks and the front of your thighs.

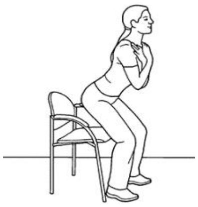

4. Sitting with your arms crossed.

Stand up and then sit down slowly on a chair. (This can be made easier by using a higher chair, and using your arms to help if you need to. This can be made harder by using a lower chair.)

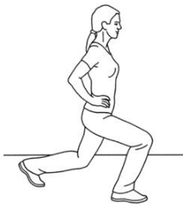

5. Stand straight. Take a step forwards and take the weight onto the front leg. Push off your front leg bringing your legs back together. Repeat with the other leg.

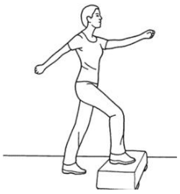

6. Stand in front of a 20 - 40 cm step.

Step up with one leg leading and then repeat with the other leg leading.

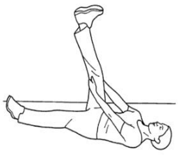

7. On your bed, lying on your back. Lift your leg towards your chest. Place your hands behind the knee. Gently pull your leg towards your chest. Feel the stretch behind your thigh. Hold for 10-30 seconds.

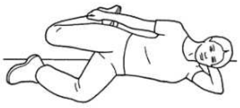

8. On your bed, lying on your side with your legs bent. Take hold of the ankle of your upper leg. Gently draw your foot towards your buttock. Feel the stretch in the front of your thigh. Hold for 10-30 seconds to the point of tightness or slight discomfort.

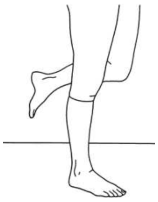

9. Stand on one leg. Try and keep your balance for approximately 10-30 seconds, or even up to 1 minute if you can. (This can be made easier by holding your arms out to the side. This can be made harder by crossing your arms, or shutting your eyes.)
