# Supplementary material for: Clinical and cost-effectiveness of bracing in symptomatic knee osteoarthritis management: protocol for a multicentre, primary care, randomised, parallel-group, superiority trial
Source: BMJ Open. 2021 Mar 26;11(3):e048196. doi: 10.1136/bmjopen-2020-048196 (PMC8006841; doi:10.1136/bmjopen-2020-048196)
Supplement: Reviewer comments [file bmjopen-2020-048196.reviewer_comments.pdf]

PEER REVIEW HISTORY

BMJ Open publishes all reviews undertaken for accepted manuscripts. Reviewers are asked to complete a checklist review form (**http://bmjopen.bmj.com/site/about/resources/checklist.pdf**) and are provided with free text boxes to elaborate on their assessment. These free text comments are reproduced below.

ARTICLE DETAILS

**TITLE (PROVISIONAL)**	Clinical and cost-effectiveness of bracing in symptomatic knee osteoarthritis management: protocol for a multi-centre, primary care, randomised, parallel-group, superiority trial
**AUTHORS**	Holden, Melanie; Callaghan, Michael; Felson, David; Birrell, Fraser; Nicholls, Elaine; Jowett, Sue; Kigozi, J; McBeth, John; Borrelli, Belinda; Jinks, Clare; Foster, Nadine; Dziedzic, Krysia; Mallen, Christian; Ingram, Carol; Sutton, Alan; Lawton, Sarah; Halliday, Nicola; Hartshorne, Liz; Williams, Helen; Browell, Rachel; Hudson, Hannah; Marshall, Michelle; Sowden, Gail; Herron, Dan; Asamane, Evans; Peat, George

This article was not externally reviewed at BMJ Open. Protocols that have been independently assessed prior to submission to BMJ Open are usually fast-tracked to publication on the grounds that further substantial changes will not be possible. This independent assessment will usually be external, independent review for both a substantial grant award from a non-commercial or government funder and ethics approval.
